# The Ribosomal Protein L28 Gene Induces Sorafenib Resistance in Hepatocellular Carcinoma

**DOI:** 10.3389/fonc.2021.685694

**Published:** 2021-07-08

**Authors:** Yi Shi, Xiaojiang Wang, Qiong Zhu, Gang Chen

**Affiliations:** ^1^ Departments of Molecular Pathology, Fujian Cancer Hospital, Fujian Medical University Cancer Hospital, Fuzhou, China; ^2^ The School of Basic Medical Sciences, Fujian Medical University, Fuzhou, China; ^3^ Departments of Pathology, Fujian Cancer Hospital, Fujian Medical University Cancer Hospital, Fuzhou, China

**Keywords:** ribosomal protein L28 gene, hepatocellular carcinoma, sorafenib, drug resistance, molecular mechanism

## Abstract

**Background:**

Sorafenib is the first molecular-targeted drug for the treatment of advanced hepatocellular carcinoma (HCC). However, its treatment efficiency decreases after a short period of time because of the development of drug resistance. This study investigates the role of key genes in regulating sorafenib-resistance and elucidates the mechanism of drug resistance in hepatocellular carcinoma.

**Methods:**

The HCC HepG2 cells were used to generate a sorafenib-resistant cell model by culturing the cells in gradually increasing concentration of sorafenib. RNA microarray was applied to profile gene expression and screen key genes associated with sorafenib resistance. Specific targets were knockdown in sorafenib-resistant HepG2 cells for functional studies. The HCC model was established in ACI rats using Morris hepatoma3924A cells to validate selected genes associated with sorafenib resistance *in vivo*.

**Results:**

The HepG2 sorafenib-resistant cell model was successfully established. The IC_50_ of sorafenib was 9.988μM in HepG2 sorafenib-resistant cells. A total of 35 up-regulated genes were detected by expression profile chip. High-content screening technology was used and a potential drug-resistance related gene *RPL28* was filtered out. After knocking down *RPL28* in HepG2 sorafenib-resistant cells, the results of cell proliferation and apoptosis illustrated that *RPL28* is the key gene involving in drug resistance. Furthermore, it was found that both RNA and protein expression of *RPL28* increased in HepG2 sorafenib-resistant specimens of Morris Hepatoma rats. In addition, the expression of proliferative protein Ki-67 increased in sorafenib-resistant cells.

**Conclusion:**

Our study suggested that *RPL28* is a key gene inducing sorafenib resistance in HCC and could be a potential target for the treatment of drug-resistant HCC.

## Background

In recent years, a number of tyrosine kinase inhibitor drugs, such as sorafenib, regorafenib, and lenvatinib have been approved in China and have become the main treatment options for patients with advanced liver cancer. Sorafenib is the first molecular-targeted drug approved to treat advanced hepatocellular carcinoma (HCC). Sorafenib, named as Dodgemet in the market, targets RAF kinase, VEGFR1-3 and PDGF receptors. Shingina et al. and Kudo et al. reported that the median survival time, disease control rate, median imaging progression time and overall survival time are better in patients treated with sorafenib than those treated with placebo (1). Chung et al. reported that HCC patients treated with sorafenib combined with TACE achieved similar results in the Chinese subgroup (2). Regorafenib is another molecular-targeted tyrosine kinase inhibitor. LeBerre et al. confirmed that regorafenib could extend the median overall survival time by 2.8 months and the disease control rate reaching 65% in HCC patients treated with sorafenib (3). Currently, several cancer treatment guidelines have been updated: regorafenib could be used to treat advanced liver cancer after sorafenib treatment. Lenvatinib is the latest oral tyrosine poly-kinase inhibitor. Kudo et al. compared the therapeutic effect of lenfatinib and sorafenib as the first-line treatment regimen for inoperable liver cancer patients. They found that the median overall survival time and objective response rate of lenfatinib is significantly better than that of sorafenib (4), suggesting that lenfatinib might be a new first-line treatment option for patients with advanced HCC.

Although these kinases inhibitor drugs bring hopes for liver cancer patients, there are also problems in clinical application. In HCC patients who have severe chronic liver disease, the impact of survival benefits has not been confirmed due to drug-induced liver toxicity (5, 6). Furthermore, there are other challenges such as lack of effective biomarkers to predict drug efficacy, low objective drug response rate, non-significant tumor burden reduction and poor quality of life of patients (7). In addition, most patients develop drug resistance during the treatment process. Although sorafenib was proved to be a well-established molecular targeted drug for HCC therapy, the cancer inhibition efficiency decreases in most patients after an average of 17.6 weeks and the mechanism of drug resistance is not clear. Because sorafenib, regarafenib and lenvatinib are multiple-target drugs, their mechanism of action involves multiple signaling pathways and involves both tumor cells and tumor microenvironment, therefore, their drug resistance mechanisms are complex (8). Due to the high heterogenicity of HCC, the same tumor tissue might have different HCC cell subsets. Different sorafenib resistance mechanisms might occur in the same patient during treatment process and effective molecular biomarkers are needed in clinical application.

Ribosomal protein L28 (*RPL28)* gene is located on human chromosome 19q13.42 and encode the 60S large subunit components of ribosomal protein L28E family (9). Ribosome is the place for protein synthesis, involving the basic functions of protein synthesis (10). Fan et al. reported that silencing ribosomal protein L28 could inhibit the proliferation and invasion of esophageal cancer cells (11, 12). Li et al. reported that the growth of human pancreatic cancer cells could be inhibited by RNA interference down-regulated ribosomal protein L39 (13). Hide et al, observed the difference in the expression of *RPL28* in colorectal cancer and adjacent tissues, but no relevance was found between the expression of L28 and development of colorectal cancer (14, 15).

This study aims to investigate the cause of sorafenib resistance. We found that *RPL28* is a key gene in regulating sorafenib resistance in HCC.

## Materials and Methods

### Establishing the Sorafenib Resistant Cell Model of Hepatocellular Carcinoma

We established the sorafenib (Selleck) drug resistant HepG2 cell model by culturing HepG2 cells with gradually increasing concentrations of sorafenib. Before the experiment, cell viability assay was conducted by Cell Titer-Glo Luminescent (Promega) and IC50 of sorafenib in HepG2 cells was selected as the starting drug concentration. Then, cells were maintained for one week until they grew steadily. After that, increased concentration of sorafenib (0.25 mol/L each time) was added to the culture medium. The induction process lasted until the cells could grow steadily at the maximum tolerable concentration of sorafenib, which took almost a year. Finally, the sorafenib resistant cell model of HepG2 was successfully constructed. The IC50 of HepG2 cells and sorafenib resistant cells were calculated by Graph pad Prism, and the resistance index (RI) = IC50 of sorafenib resistant cells/IC50 of HepG2 cells, was calculated (16, 17).

### Screening Drug Resistance Genes

Gene chip Primeview human (Affymetrix) for expression spectrum profiling was applied to select the drug resistance-related genes. Screening criteria were fold change (FC) >2 and P-value <0.05 (18, 19). Stratagene Mx3000P real-time PCR (Agilent) was used to detect the endogenous expression of drug resistance-related genes in these cells (20). The gene RPL28 was screened out by High Celigo Select (HCS) technique to be the key gene of drug resistance (21–23).

The characteristic of drug-resistant cells is the loss of response to drug. Therefore, we observed the effect of silencing target gene on cell proliferation by down regulating the expression of target gene in lentivirus infected cells. The green fluorescent protein GFP was expressed by infected cells and counted automatically. In order to ensure the gene interference efficiency, we designed three RNA interference targets for each gene, and the three plasmids were mixed as equal proportion and then packaged with lentivirus. Cells were photographed and automatically counted by the Celigo fluorescence microscope (Olympus). The influence of the cell proliferation for each gene was detected by the high throughput model. Data were expressed as mean ± standard deviation (SD) and Graph pad Prism software was applied to perform statistical analysis. Different expression of gene between HepG2 cells and sorafenib resistant cells were evaluated using a paired t test. The p value less than 0.05 was considered to be of statistical significance.

By the same method, we screen out the target with the most significant inhibition of cell proliferation and the highest knockout efficiency of key resistance gene. After determining the proliferation-related positive target genes, lentivirus packaging and HCS cell proliferation detection were performed on the plasmids of the three RPL28 RNA interference targets, to further confirm the specific targets of the gene affecting cell proliferation.

Using RPL28-1 target as a template (**Table 1**), we prepared RNA interference lentivirus vectors and infected HepG2 sorafenib resistant cells. The reduced protein expression of RPL28 gene was detected by Western blot (24). The effect of RPL28 gene on the proliferation, apoptosis and cell cycle of HepG2 sorafenib resistant cells were detected by Celigo (Nexcelom) (25), Caspase-Glo^®^ 3/7 Assay (Promega), Apoptosis kit (BD) and PI (Sigma) (26). GAPDH was used as reference gene to standardize the expression of each target. Using relative quantitative analysis (F= 2- ΔΔCT), the relative expression level of RPL28 were calculated. Difference of proliferation, apoptosis and cell cycle between HepG2 cells and sorafenib resistant cells were evaluated using a paired t test. The P value less than 0.05 was considered of statistical significance.

**Table 1:** RNAi target sequence.

Gene targets	Sequences
*RPL28*-1	CTACAGCACTGAGCCCAATAA
*RPL28*-2	TGGTGGTCATTAAGCGGAGAT
*RPL28*-3	CCGCAATTCCTTCCGCTACAA

### Rat Model for Sorafenib Resistance

SPF rats were used to establish Morris hepatoma liver cancer model. Morris hepatoma 3924A (MH3924A) cells were subcutaneously injected and about 1×1×1cm^3^ tumor could be touchable in the subcutaneous in about 2 weeks. Under aseptic conditions, the tumor bulk was inoculated to the left lobe of the liver of ACI rats to create liver carcinoma model in situ (27, 28). After treatment with sorafenib, dynamic change of rat tumor in sensitive period and resistance period of sorafenib were detected by PET-CT. The expression of *RPL28* gene in sorafenib-resistant HCC was tested by Real time qPCR. IHC was used to detect the expression of KI-67 in samples in both sorafenib sensitive and resistance stage. The intensity and the proportion of stained cells were integrated, and the result was determined by semi-quantitative integral method using the following scoring criteria: staining intensity score, 0=no staining, 1= weak staining, 2 = moderate staining, 3= strong staining; staining percentage score, 0= positive percentage<10%, 1 = 10-25%, 2 = 26-50%, 3 = 51-75%, 4> 75%. The staining score = intensity score x percentage. The score of 0-3 indicates low expression, while 4-7 indicates high expression.

### Statistical Analysis

Data were presented as mean ± SD. Graph pad Prism software was applied to perform statistical analysis. Difference between groups was evaluated using a paired t test. The p value less than 0.05 was considered to be of statistical significance.

## Results

### Testing the Sorafenib Resistant Cell Line

**Figure 1:**
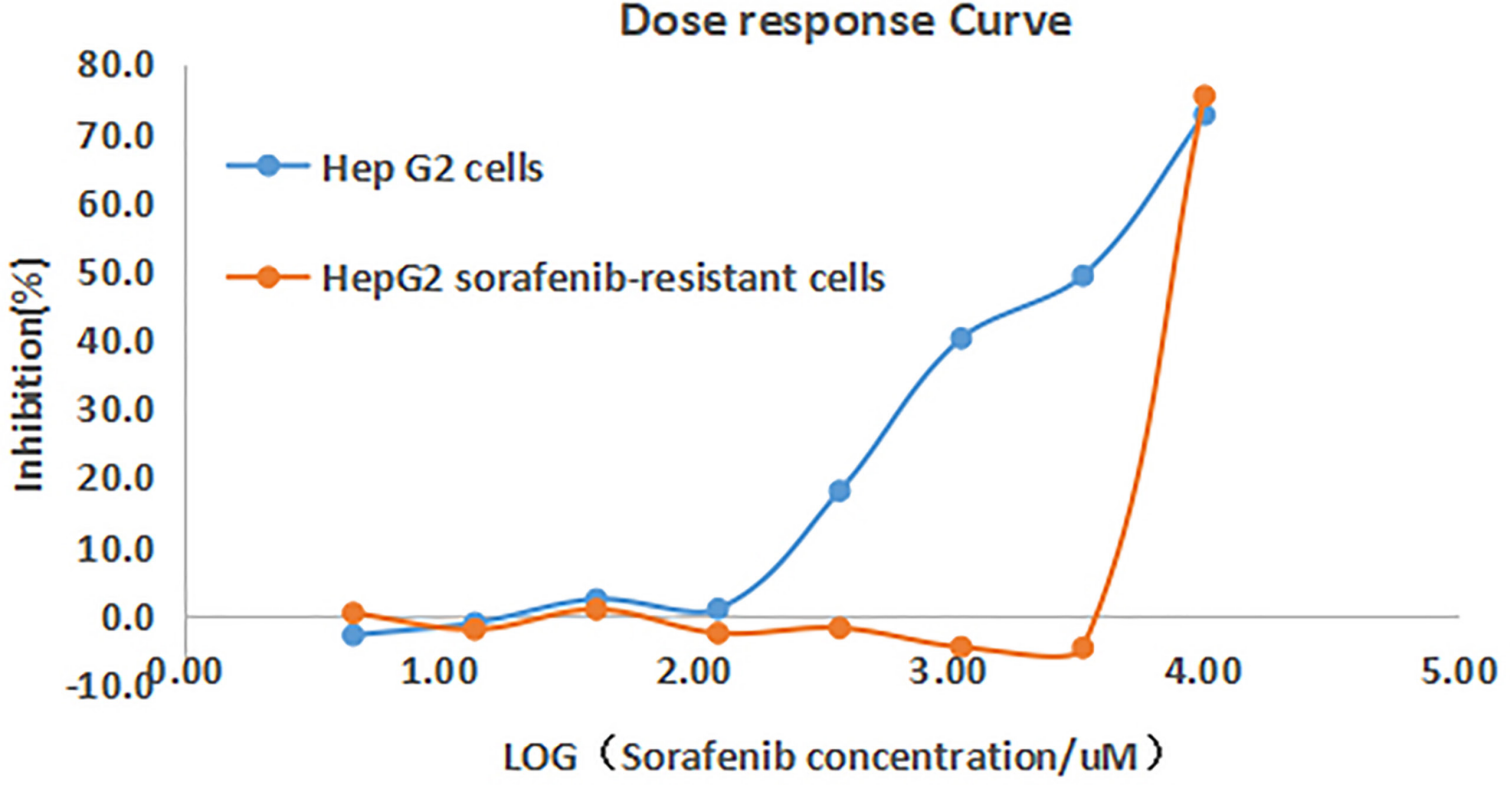
Inhibition curves of sorafenib on HepG2 and HepG2 drug-resistant cells. The inhibitory rate of HepG2 cells increased with the increasing drug concentrations, while no significant change on inhibitory rate of HepG2 drug-resistant cells.

The cell proliferation in response to increasing concentrations of sorafenib was examined in both HepG2 control and HepG2 drug resistant cells. It was found that the inhibitory rate of HepG2 cells increased significantly with the increasing drug concentrations, with the IC_50_ of sorafenib being 1.62μM in control cells, while no significant change on inhibitory rate in HepG2 drug-resistant cells, and the IC_50_ was 9.988μM. The drug resistance fold was 5.97 (RI=IC50 = 9.988/1.672 = 5.97). This result indicated the successful establishment of sorafenib resistant cell model (**Figure 1**).

### Detecting Drug Resistant Genes

**Figure 2:**
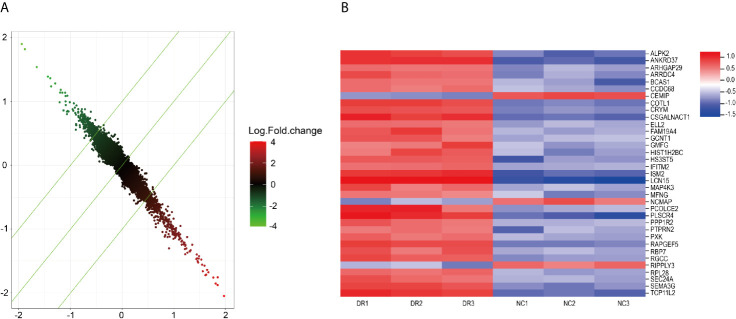
**(A)** Drug-resistant related genes detected by microarray. **(B)** important genes related to drug-resistance.

Gene expression profile chip was used to screen genes related to drug resistance. 295 up-regulated genes and 211 down-regulated genes were found in HepG2 sorafenib resistant cells compared with HepG2 control cells (**Figure 2**). Through the bioinformatics analysis, 35 up-regulated genes were selected for further study based on their highly expressed profiles in drug-resistant cells.

**Figure 3:**
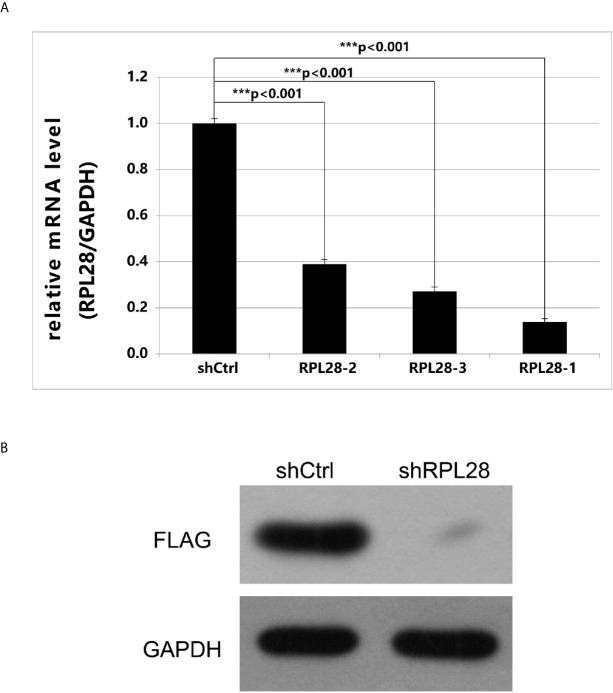
**(A)** Expression of RPL28 gene after it was knocked down. The expression of *RPL28* genes in HepG2 drug-resistant cells was 0.139,0.389 and 0.272, compared with control group, after *RPL28*-1, *RPL28*-2 and *RPL28*-3 target was knocked down, respectively. The differences were statistically significant (P < 0.05). **(B)** Western blot on protein expression after shRNA knockdown of *RPL28*.

Among the 35 drug-resistant related genes, two genes were not detected by qPCR due to difficulty in primer design, and one gene had low expression. The remaining 32 drug-resistant related genes had high abundance of expression. Twenty genes were randomly selected based on the screening condition with proliferation change ≥2.0, and the difference significance analysis T-test value <0.05. The key drug-resistant gene *RPL28* was selected by comparing the effects of gene knockdown on cell proliferation using HCS technology. RPL28-1 was finally selected as the key drug-resistant gene. After knocking-down of RPL28-1, the proliferation inhibition of HepG2 drug-resistant cells reached 2.76 times compared with the control cells, and the knockdown efficiency reached 86.1% compared with the control cells (**Figure 3A**), and the difference was statistically significant (P <0.05), indicating that *RPL28*-1 was indeed an effective target.

### Preparing RNAi Lentiviral Vector of RPL28 Gene and RPL28 Knockdown Affected Protein Expression

Using *RPL28* gene as a template, RNA interference target sequence was designed, and RNA interfering lentivirus vector was constructed. The sequence of *RPL28* gene interference target in the vector starts from the C of 383bp and ends at A of 403bp. HepG2 drug-resistant cells were infected by lentiviruses with infection efficiency of above 80%, indicating that the RPL28 knockdown sorafenib resistant HepG2 cell line was successfully established. Western Blot was applied to detect the expression of exogenous protein of *RPL28* gene and we found that knockdown the *RPL28*-1 target downregulated the exogenous expression of *RPL28* gene (**Figure 3B**), which further proved that *RPL28*-1 was an effective interference target.

### Biological Function Study of HepG2 Sorafenib Resistant Cells Infected by shRNA Lentivirus

**Figure 4:**
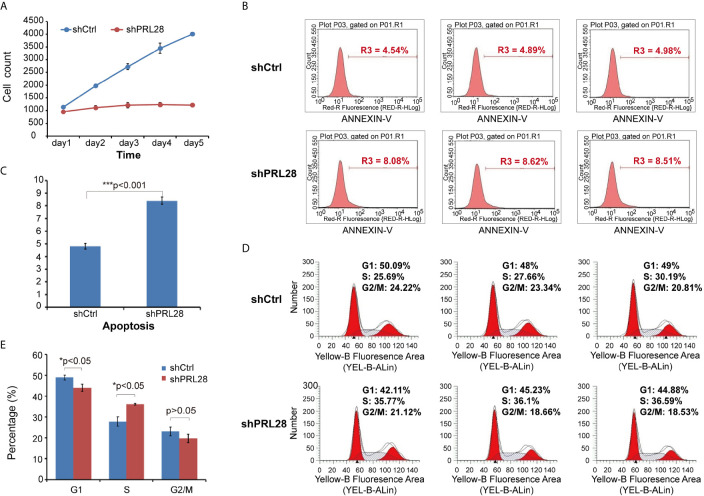
Cell counting, apoptosis and cell cycle analysis on shRNA lentivirus infected HepG2 sorafenib resistant cells. **(A)** Cell counting result showed the proliferation of shRPL28 and shCtrl cells. **(B, C)** Peak diagram and histogram of cell apoptosis (X ± S): The apoptotic rate of shRPL28 cells was higher than that of shCtrl cells. **(D, E)** Cell cycle analysis result of G1, S and G2/M phase.

After knocking- down of *RPL28* gene, the proliferation of sorafenib resistant HepG2 cells was significantly inhibited as tested by Celigo. Cell counting result showed that the proliferation of sh*RPL28* cells was significantly inhibited compared with the shCtrl cells (**Figure 4A**). Caspase-Glo^®^ 3/7 Assay and flow cytometric analysis showed that the apoptosis rate of sorafenib resistant HepG2 cells increased when *RPL28* gene was knockdown (**Figures 4B, C**). The results of cell cycle analysis illustrated that the cells in S phase increased after knocking-down *RPL28* in HepG2 sorafenib resistant cells and decrease the cell number in G1 phase. Our results showed the strong association between the function of *RPL28* and cell cycle change in HepG2 sorafenib resistant cells (**Figures 4D, E**).

### *In Vivo* Evaluation for Sorafenib Resistance

**Figure 5:**
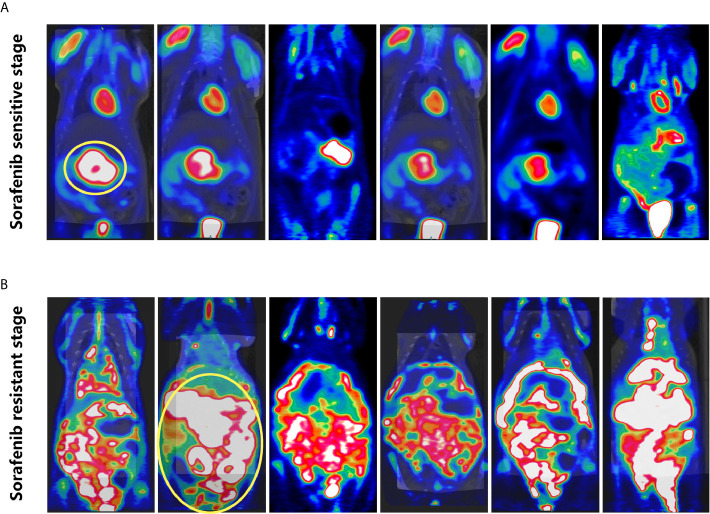
PET-CT of rats. **(A)** Sorafenib sensitive stage: tumor growth was restricted. **(B)** Sorafenib resistant stage: the tumor was widely metastasized to the abdominal cavity.

Dynamic changes of tumor in rat with sorafenib treatment were evaluated by PET-CT. The tumor was controlled in the sorafenib sensitive stage (**Figure 5A**), however, during sorafenib resistant stage, the tumor can be seen extensively metastasis to abdominal cavity (**Figure 5B**).

**Figure 6:**
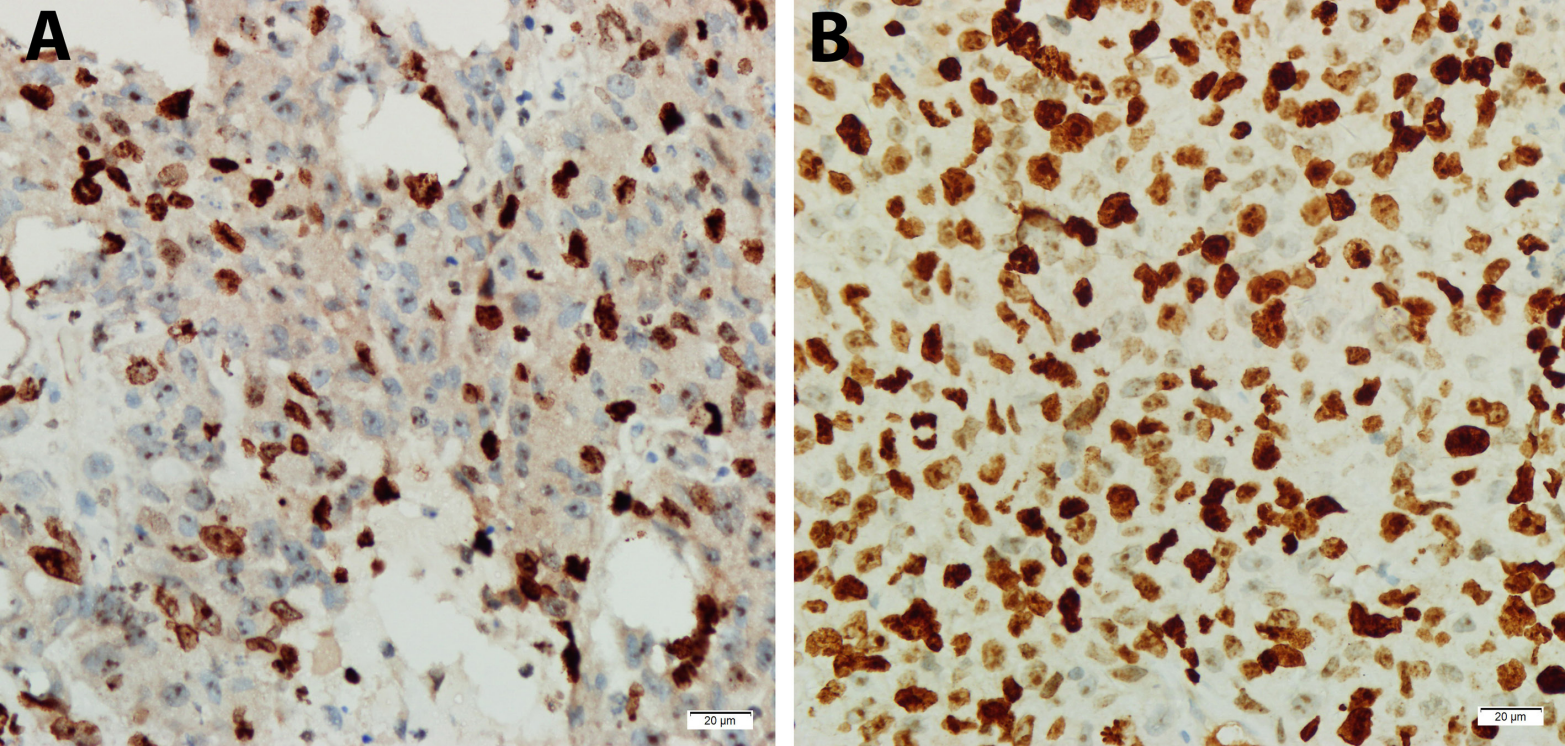
Expression of Ki67 in tumor specimens of sorafenib sensitive and resistant stage by immunohistochemistry (DAB developing, 400X). **(A)** Sorafenib sensitive period, Ki-67 protein (Localization in nucleus) with low expression. **(B)** Sorafenib resistance period, Ki-67 protein with high expression.

The expression of sorafenib-resistant associated gene *RPL28* in Morris Hepatoma was determined by real time qPCR. The expression of *RPL28* increased in the sorafenib-resistant specimens compared with that in sensitive phase with statistically significant (P<0.05), indicating that *RPL28* gene was associated with sorafenib resistance. The expression of Ki-67 in HCC tissues of rats with sorafenib sensitive stage was lower than that of rats with drug resistance stage, indicating that sorafenib inhibited the proliferation of HCC cells (**Figures 6A, B**).

## Discussion

At present, there are two main ways to establish drug-resistant tumor cells *in vitro*: drug induction and drug resistant gene transfection. The drug-resistant cell lines established by drug induction method is similar to the development of drug resistance in clinical patients, which is more suitable for the experimental model of drug resistance research of tumor cells. Drug induction methods include high dose intermittent impact method, concentration gradient increasing method, and a combination of the two methods. Among them, the first two methods are often used to establish the drug resistance model of clinical tumor cells and each has its own advantages and disadvantages. The high dose intermittent impact method is suitable for the establishment of drug resistance cell model of chemotherapy drugs, which is close to the principle of chemotherapy in the clinical high dose and short course of chemotherapy. But the disadvantage is that the drug concentration of high dose intermittent impact method is very high, and the cells may be difficult to tolerate due to the sudden changes of external environment. The advantage of the concentration gradient method is that the drug dose of induced cells is gradually changed, the external environment of cell culture is gradually changed, and the cells are easy to accept and in better condition. Sorafenib is a small molecule targeted drug that requires long-term oral administration to maintain the concentration of blood drug, so it is more suitable to establish drug-resistant cell models by this method.

In this study, a total of 35 up-regulated drug-resistance related genes were screened by Affymetrix expression profile chip for subsequent studies. Using Real Time qPCR technology, we verified that 32 genes were highly expressed in HepG2 sorafenib resistant cells. Among the 32 genes with high expression, 20 genes were randomly selected to detect their effect on cell proliferation, so as to screen out the key genes related to sorafenib resistance. RPL28, a key gene related to sorafenib resistance, was screened out from the above 20 genes. We performed single target lentivirus amplification and proliferation detection of RPL28 to further confirm the specific target of the gene affecting the proliferation function.

The RPL28 gene is the typical gene which encodes ribosomal protein with many pseudo-genes scattered throughout the genome and different splicing transcription forms. In this study, the expression of *RPL28* gene was found to be significantly increased in sorafenib resistant HepG2 cells compared with parental HepG2 cells. Ribosomes act as a processing factory of protein polypeptide chains and the expression of RP itself reflects the state of cell growth. Ribosome proliferation also indicates proliferative activity of both normal and tumor tissues. Growing cells have more efficient ability for translating RP mRNA than stationary cells. Thus, cells with proliferating activity usually have high RP levels. Therefore, when HepG2 cells developed resistance to sorafenib, the cell proliferation inhibited due to drug action became active again, and the expression of ribosomal protein increased accordingly, which could explain our experimental results.

In order to verify the experimental results, we constructed a *RPL28* RNA interference lentiviral vector, and the HepG2 sorafenib resistant cells were infected with the lentivirus to down-regulate the *RPL28* gene expression. It was found that knocked out of *RPL28* gene significantly inhibited the proliferation of HepG2 sorafenib resistant cells and increased apoptotic cells. Our results suggested that interfering *RPL28* gene could inhibit the proliferation of HepG2 sorafenib resistant cells, reversing sorafenib resistance. Therefore, up-regulation of *RPL28* gene might be one of the causes of sorafenib resistance, and its mechanism is associated with the effect of RP in tumors. Changes in RP expression may affect the protein translation process. Both the assembly and function of ribosomes depend on the balance of the quantity of RP. The increased ribosomes may accelerate the translation of ribosomes and affect the synthesis of proteins that affect the metabolism and biological functions of cells. We speculated that the increased *RPL28* gene expression would accelerate mRNA translation and protein synthesis, promote HepG2 cell proliferation, and lead to sorafenib resistance.

Ribosomal proteins can regulate cell apoptosis. Zhang et al. reported that RP S15A was highly expressed in glioma compared with normal tissues. Down-regulation of RPS15A could induce apoptosis of glioma cells (29). Our results indicated that the increased expression of *RPL28* gene might down-regulate certain apoptotic proteins, inhibit the programmed cell death of HepG2 cells, and result in sorafenib resistance. The most reported function of ribosomal protein is related to tumor suppressor gene P53. Peng et al. reported that ribosomal protein L23 inhibit P53 ubiquitination by negatively regulating murine double minute 2 (MDM2), thereby activating P53 to inhibit tumor growth (30, 31). It might be possible that the increased expression of *RPL28* gene also regulate the P53 gene, and lead to sorafenib resistance.

Morris hepatoma3924A is a highly metastatic cell strain of rat liver cancer. Due to its high tumor formation rate *in vivo*, it has been widely used to establish various animal models. Jun et al. applied Morris hepatoma3924A in ACI rat to explore nanoscale integrin combined with arterial chemoembolization for the treatment of liver carcinoma (32). In this study, Morris hepatoma3924A in ACI rat model was used to study sorafenib resistance *in vivo*. It was found that the tumor growth was effectively controlled in the sorafenib sensitive stage. The tumor had extensive metastasis in the abdominal cavity in the sorafenib resistant stage.

Real time qPCR and IHC results showed that the protein expression of *RPL28* gene increased in the resistant phase compared with the sensitive phase, which is in consistent with the results *in vitro*, and indicating that *RPL28* gene might be the sorafenib-resistant gene *in vivo*. In addition, immunohistochemical staining results showed that Ki-67 protein expression increased in the drug-resistant stage compared to the sensitive stage. Ki-67 is used to judge cell proliferative activity. It is expressed in all cell cycles (G1, S, G2 and M stages), but not expressed in G0 stage. Previous studies indicated that the KI-67 expression is closely associated with cell proliferation, differentiation, invasion and metastasis, and prognosis of various tumors. In this study, ki-67 protein expression increased in sorafenib resistance rats, suggesting that sorafenib resistance is related to cell proliferation. The cell cycle regulatory core molecule is CDK, which is dependent on the expression of Cyclin. CDK4 is one of the CDK series of protein kinases. CDK4 kinase activity is activated when CDK4 binds to periodic protein D1 (cyclin D1) to form the CDK4/cyclin D1 complex, which phosphorylates Retinoblastoma protein (Rb). Rb protein releases a transcription factor E2F, results in the expression of S phase related proteins induced by E2F. It promotes cells to complete DNA replication, quickly across the G1 to S phase limit point, stimulate cell growth and division (33). Over-expression of CDK4 caused HepG2 cells growth out of control, promoted cell proliferation and abnormal differentiation leading to sorafenib resistance. Bcl-2 (B-cell lymphoma 2 gene) is an oncogene. Its family of proteins play an important role in mitochondrial apoptosis. The Bcl-2 protein family has high homology and contains BH (1-4) conserved domains. The function of Bcl-2 protein family can be divided into two categories, one is anti-apoptotic: including Bcl-2, Bcl-XL and McL-1, that are located in the mitochondrial outer membrane, endoplasmic reticulum and nuclear membrane. The other is pro-apoptotic, including Bax, Bak and Bid. They are mostly located in the cytoplasm (34).

## Conclusion

In conclusion, this study established a sorafenib resistant hepatocellular carcinoma cell model. The drug resistant gene was screened by expression profile chip technology and confirmed by experiments both *in vitro* and *in vivo*. We identified that *RPL28* gene is involved in sorafenib resistance in hepatocellular carcinoma. Our study provides a new approach to overcome sorafenib resistance in the treatment of hepatocellular carcinoma.

## Data Availability Statement

The original contributions presented in the study are included in the article/supplementary material. Further inquiries can be directed to the corresponding author.

## Ethics Statement

This study was approved by the Fujian Medical University Cancer Hospital. Written informed consent for participation was not required for this study in accordance with the national legislation and the institutional requirements.

## Author Contributions

GC conceived and designed the study. YS, XW, and QZ performed experiments and analyzed results. YS wrote the first draft. GC edited the manuscript. All authors contributed to the article and approved the submitted version.

## Funding

This research was supported by the Natural Science Foundation of Fujian Province, No. 2018J01268; Fujian Provincial Health Technology Project, No. 2018-ZQN-15; and Science and Technology Program of Fujian Province, China, Nos. 2018Y2003, 2019L3018, and 2019YZ016006.

## Conflict of Interest

The authors declare that the research was conducted in the absence of any commercial or financial relationships that could be construed as a potential conflict of interest.

## Abbreviations

Bcl-2, B-cell lymphoma 2; GFP, green fluorescent protein; HCC, hepatocellular carcinoma; MH3924A, Morris hepatoma 3924A; RPL28, Ribosomal protein L28; HCS, High Celigo Select; PCR, polymerase chain reaction.
